# The LINC complex, mechanotransduction, and mesenchymal stem cell function and fate

**DOI:** 10.1186/s13036-019-0197-9

**Published:** 2019-08-07

**Authors:** Tasneem Bouzid, Eunju Kim, Brandon D. Riehl, Amir Monemian Esfahani, Jordan Rosenbohm, Ruiguo Yang, Bin Duan, Jung Yul Lim

**Affiliations:** 1 Department of Mechanical and Materials Engineering, University of Nebraska-Lincoln, W317.3 Nebraska Hall, Lincoln, NE 68588 USA; 2 Mary & Dick Holland Regenerative Medicine Program, University of Nebraska Medical Center, Omaha, NE USA; 3 Division of Cardiology, Department of Internal Medicine, University of Nebraska Medical Center, Omaha, NE USA

**Keywords:** Mesenchymal stem cells, Linker of Nucleoskeleton and cytoskeleton (LINC), Nesprin, SUN, Mechanotransduction, Functional tissue engineering

## Abstract

Mesenchymal stem cells (MSCs) show tremendous promise as a cell source for tissue engineering and regenerative medicine, and are understood to be mechanosensitive to external mechanical environments. In recent years, increasing evidence points to nuclear envelope proteins as a key player in sensing and relaying mechanical signals in MSCs to modulate cellular form, function, and differentiation. Of particular interest is the Linker of Nucleoskeleton and Cytoskeleton (LINC) complex that includes nesprin and SUN. In this review, the way in which cells can sense external mechanical environments through an intact nuclear envelope and LINC complex proteins will be briefly described. Then, we will highlight the current body of literature on the role of the LINC complex in regulating MSC function and fate decision, without and with external mechanical loading conditions. Our review and suggested future perspective may provide a new insight into the understanding of MSC mechanobiology and related functional tissue engineering applications.

## Introduction

Cellular sensing and response to changes in extracellular environments, biochemical and mechanophysical, are critical for cell growth and function. Mechanical forces both outside and inside the cell can be transduced into molecular signaling activities to direct cellular function and fate - a process known as mechanotransduction [1]. The mechanical forces at the plasma membrane can be altered either via changes in cell-substrate adhesion due to modifications in extracellular matrix (ECM) density, rigidity, and orientation, or through the formation or dissociation of cell-cell junctions. The altered force at the plasma membrane can biochemically or mechanophysically affect membrane-bound mechanosensitive proteins, such as integrin cell-ECM adhesion and cadherin cell-cell junction proteins and linker proteins bound to them [2]. This, in turn, may induce the reorganization of cytoskeletal proteins, such as actin filaments, anchored at the cell-ECM and cell-cell adhesion junctions. Such procedures can trigger changes in related downstream molecular signaling pathways. The cytoskeleton provides a pathway for mechanical forces to be transferred from the plasma membrane to internal cellular structures, including the nucleus [3]. The nuclear-cytoplasmic connections facilitated by nuclear envelope (NE) proteins such as the Linker of Nucleoskeleton and Cytoskeleton (LINC) complex provide spatial and structural integrity for the nucleus, as well as allow for the transfer of mechanical force into the nucleus resulting in mechanotransduction [4]. In this review, considering the recent interest in the mechanotransduction community, we will first address the importance of the LINC complex and component proteins, nesprin and SUN, in regulating cellular function and fate in general. While the LINC complex has not been fully established to control mesenchymal stem cell (MSC) behavior via mechanotransduction mechanisms, we will highlight recent advances in the understanding of how the LINC complex is potentially involved in the mechanical regulation of MSC behavior, lineage commitment, and differentiation.

## LINC complex

**Fig. 1:**
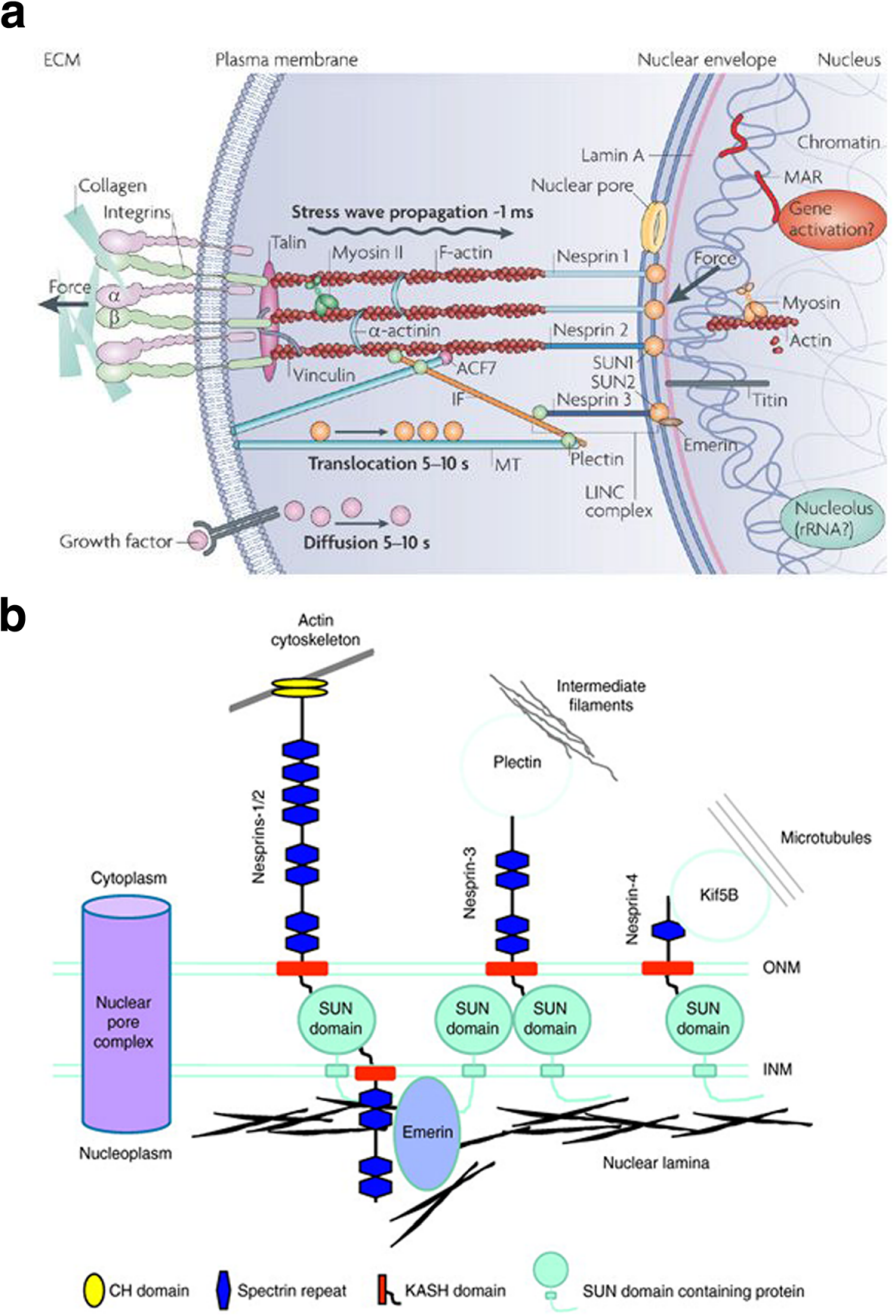
Schematic of the LINC complex including nesprin and SUN and their connections to cytoskeletal proteins. **a** External forces are localized at integrins and channeled through cytoskeletal filaments to the nucleus. Once at the nuclear surface, forces transmit through nesprins to SUN proteins which are linked to lamin proteins that form the lamina and nuclear scaffold. Lamin interacts with DNA machinery and related elements to alter gene expression. Reprinted with permission from [6]. **b** The KASH domain of nesprins at the ONM associates with the SUN domain located in the perinuclear space (PNS, not labeled) between the ONM and INM. The linking of the nuclear envelop to the cytoskeleton is shown: nesprin-1/2 with actin, nesprin-3 with intermediate filament, and nesprin-4 with microtubule (nesprin-5 is not connected to cytoskeleton). Reprinted with permission from [7]

The nuclear membrane is composed of an inner nuclear membrane (INM) and an outer nuclear membrane (ONM), which are separated by a ~ 40 nm gap known as the perinuclear space (PNS) [5]. The ONM is attached to the endoplasmic reticulum (ER), where the PNS forms a continuous extension into the ER lumen. A key feature of the nuclear envelope is the lamina, a mesh of proteins lining the inner surface of the INM. Importantly, the lamina is composed of lamin A/C (or LMNA) proteins that are mechanically connected to varying cytoskeletal proteins via the LINC complex (Fig. 1a) [6].

The LINC complex is composed of two protein domains: SUN (Sad1p, UNC-84) domain which spans the inner nuclear membrane, and the conserved C-terminal KASH (Klarsicht/ANC-1/Syne Homology) domain which spans the outer nuclear membrane. The C-terminal KASH is expressed in proteins known as nesprins (nuclear envelope spectrin-repeat proteins) [8–11], which are similar in structure to type II integral membrane proteins featuring a single transmembrane segment followed by a short luminal sequence [12]. The C-terminal KASH domain of nesprin proteins extends into the perinuclear space and interacts with the SUN domain of SUN proteins to form the LINC complex. KASH proteins have one or more complementary SUN proteins, allowing for various LINC complex isoforms to exist [5]. Importantly, variable N-terminal domains of nesprins support the binding with different cytoskeletal components. There have been four mammalian nesprins (Syne 1–4) identified, which encode a wide range of alternatively-spliced isoforms (Fig. 1b) [5, 7, 13]. Giant nesprins (nesprin-1 and nesprin-2) are the largest of the isoforms, each having a size of ~ 976 kDa and ~ 764 kDa, respectively, and contain N-terminal calponin homology (CH) domain that binds to F-actin [7]. On the other hand, nesprin-3 has an N-terminal motif that can bind to plectin, the intermediate filament (IF) linker protein, and nesprin-4 can indirectly interact with microtubules [14]. In addition to these four nesprins, KASH5, a meiosis-specific KASH domain, interacts with the dynein-dynactin complex and has a role in mediating telomere localization [15].

## LINC and cellular function

The LINC complex has recently been proposed to be implicated in various aspects of cellular organization and function. A primary function of LINC is in the control of the orientation of the nucleus. Arsenovic et al. [16] utilized a fluorescence resonance energy transfer (FRET)-based sensor to demonstrate that the LINC complex, specifically nesprin-2 giant (nesprin-2G), can sense myosin-dependent cellular tension to alter the nuclear shape. Beyond the nucleus shape, the LINC complex and related nuclear envelope-actin linkage have a potential to affect the functions of centrosomes [17] and meiotic chromosomes [18] to regulate cell division and replication. Further, nucleus architecture and resultant skeletal dynamics governed by the LINC complex may play a regulatory role in the repair of DNA damage [19–21]. For example, mouse embryonic fibroblasts, when doubly impaired in SUN1/2 genes, showed excessive DNA damage, increased genome instability, and compromised DNA repair [20]. In accordance, the UNC-84 domain of the SUN protein could contribute to promote the repair of interstrand crosslinks and inhibit nonhomologous end joining [21]. Enabled by the physical interconnection between the LINC complex and nuclear chromatin, epigenetic control of cell differentiation can also occur through mediation of transcription factors [22, 23].

**Fig. 2:**
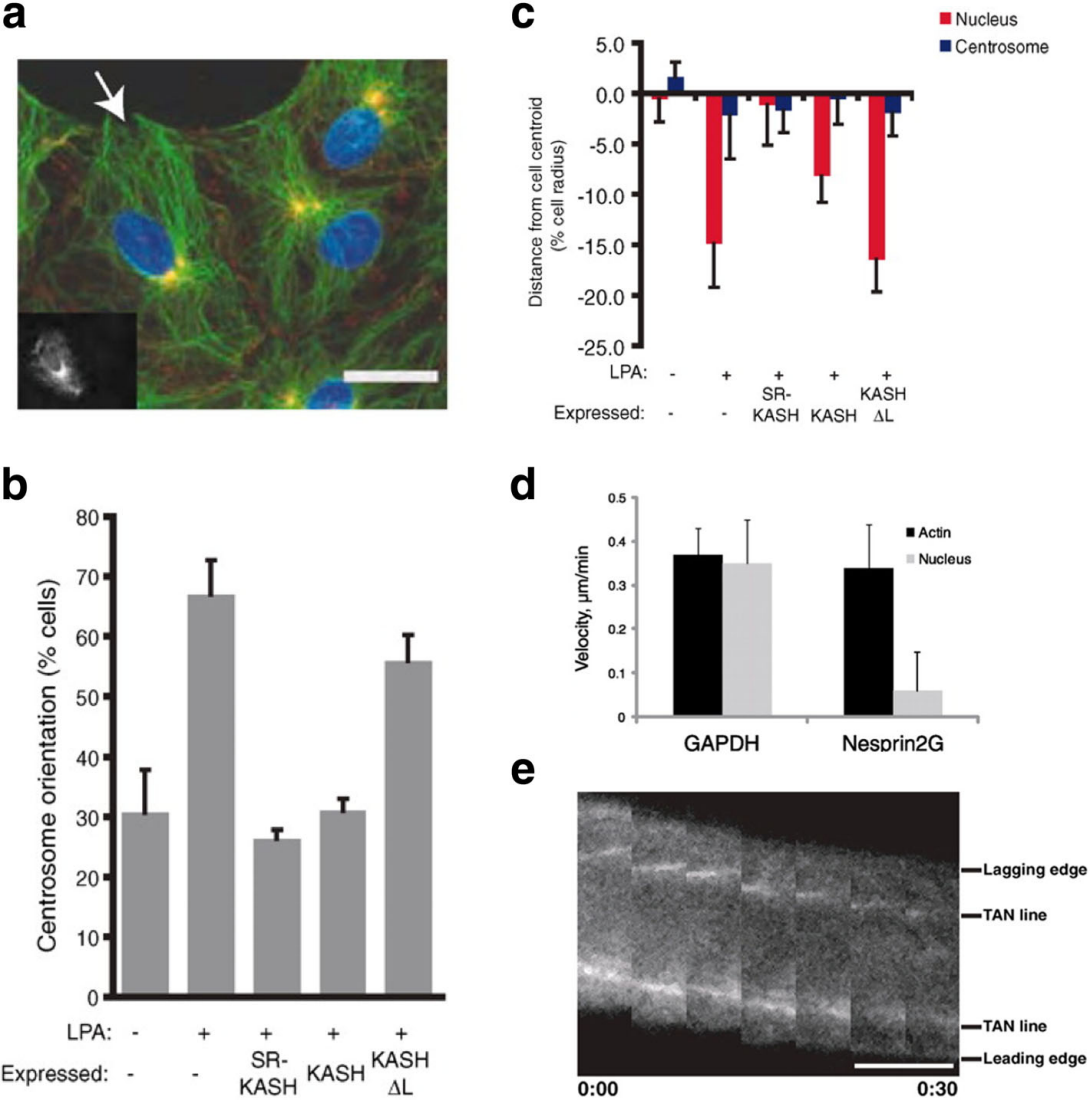
Cytokine-driven centrosome orientation requires nesprin-2G. NIH3T3 fibroblasts expressing dominant negative constructs (RFP-Spectrin Repeat-KASH, or RFP-SR-KASH, and RFP-KASH) targeting the LINC complex were stimulated with lysophosphatidic acid (LPA). **a** Epifluorescence image showing centrosome orientation of RFP-KASH-expressing cells (cell expressing RFP-KASH is indicated by arrow and in the inset). DNA (blue); centrosomes (yellow); microtubules (green); β-catenin for cell-cell adhesion (red). Scale bar = 15 μm. **b** Centrosome orientation by LPA treatment was impaired by dominant negative KASH. **c** Average centrosome and nucleus positions perpendicular to the wound (positive values are towards the leading edge; negative values are away). Nuclear movement by LPA was disabled by dominant negative KASH. **d** Nucleus velocity was decreased for nesprin2G-depleted cells. **e** Fluorescence kymograph of TAN lines in a nesprin2G-depleted nucleus during nuclear movement with time (hour:min). Scale bar = 5 μm. Adapted and reprinted with permission from [25]

Due to the coordination of nucleus morphology by the LINC complex, nucleus shape and movement during cell migration and polarization are also regulated by LINC proteins [24–26]. It was shown that nesprin-1/2 and SUN1/2 complex could couple the nucleus and centrosome during neuronal migration [24]. A study by Luxton et al. [25] utilized dominant negative constructs of the LINC complex expressed in wound-edge NIH3T3 fibroblasts to examine the role of the LINC complex in nuclear movement during migration. In their study, using lysophosphatidic acid (LPA), a motility stimulator, it was revealed that nuclear movement was significantly impaired in nesprin-2G-depleted cells (Fig. 2). Moreover, nesprin-2G and SUN2 could couple with transmembrane actin-associated nuclear (TAN) lines to assemble the nucleus to the actin cytoskeleton, thus enabling nuclear migration during cell polarization and centrosome reorientation. The proposed role of the TAN lines may parallel that of the focal adhesion complex considering that both assemble in reaction to actin bundling and both can transmit forces across membranes.

Considering its interactive role in bridging the cytoskeleton with the nuclear lamina, the LINC complex has been associated with a range of diseases [27]. Particularly, laminopathies or mutations in lamin A/C genes result in a variety of tissue-specific disorders including, but not limited to, Emery-Dreifuss muscular dystrophy (EDMD) and limb girdle muscular dystrophy 1B. A majority of lamin A/C mutations can produce cardiac and skeletal muscle defects: in skeletal muscle, such mutations disorganize the LINC complex in synaptic nuclei, leading to the dislocation of nuclei at the neuromuscular junction. Syne mutations have also been associated with non-muscular diseases including cerebellar ataxia and autosomal recessive arthrogryposis. Moreover, it was reported that mutations in nesprin-1, nesprin-2, and lamin A/C were found in a genome-wide screening of 100 breast cancer patients [28]. Similarly, mutations in nesprin-1 were observed in patients with breast, ovarian, or colorectal cancers [29], and a downregulation or mutation in the nesprin gene could be linked to an increased risk of invasive ovarian cancer [30]. Observations suggest that improved understanding of the role that the LINC complex has in cell functions may lead to the development of targeted therapies for various diseases, including a wide range of laminopathies and cancers.

## Sensing mechanical environments through the LINC complex

Targeted studies have been conducted to reveal the role of an intact nuclear envelope in mediating the ability of cells to sense and respond to extracellular mechanical environments. It was observed that under lower levels of fluid flow-induced shear stress, nesprin-2 and lamin A expression in endothelial cells were suppressed (relative to normal shear stress counterparts), leading to an increase in both cell proliferation and apoptosis [31]. Nesprin-3 was found to play a vital role in fluid shear-induced polarization of the centrosome and directional migration of human aortic endothelial cells [32]. For fibroblasts and myoblasts under fluid shear, the perinuclear actin cap, a subset of actin filaments connected to the nuclear envelope through nesprin-2G and nesprin-3, showed a dominant response to lower shear stresses, while conventional actin filaments at the basal surface of the cells required at least 50 times more shear stress to assemble [33].

**Fig. 3:**
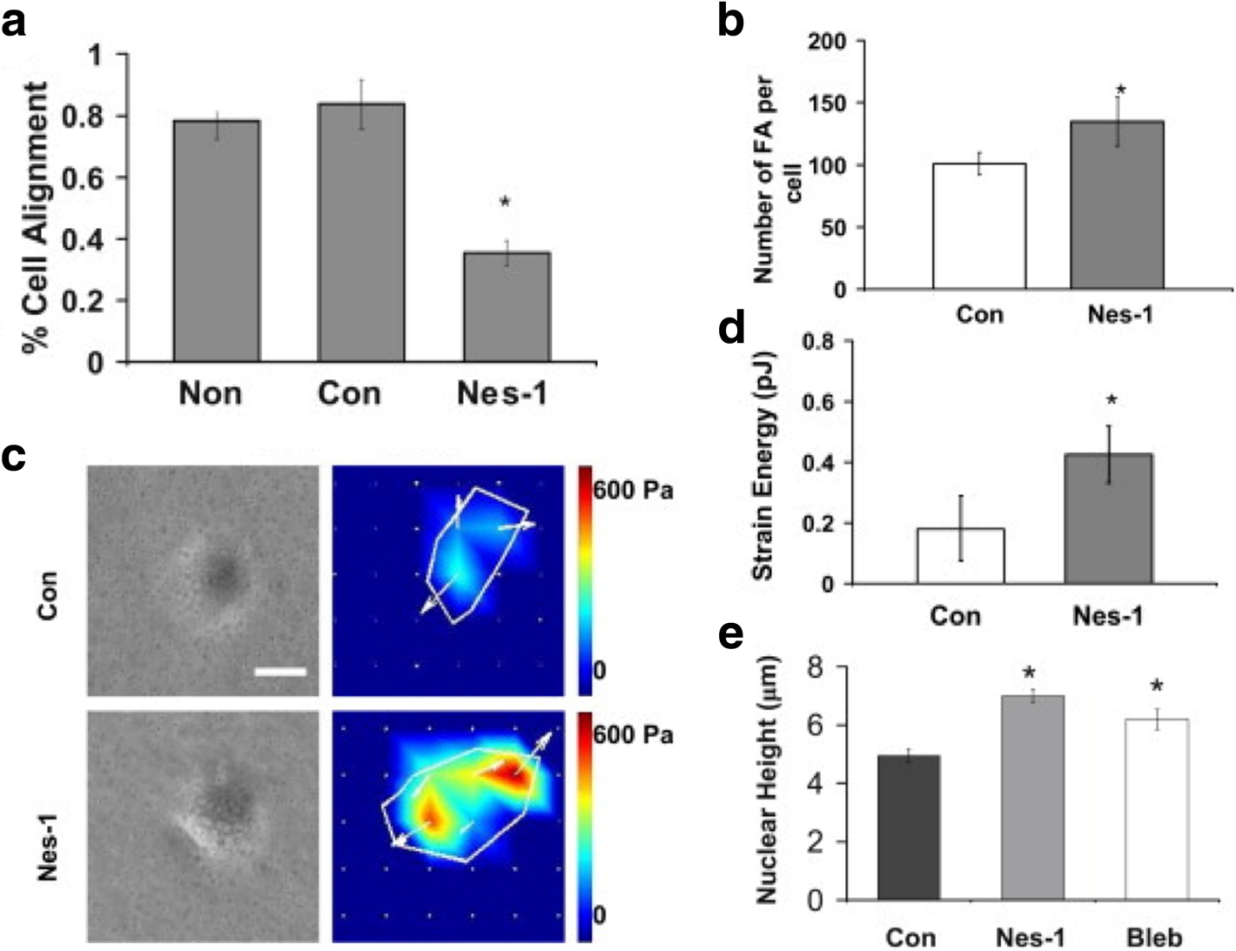
Nesprin-1 deficient vascular endothelial cells are unable to align in response to cyclic strain. **a** HUVECs seeded on silicon membranes were exposed to 10% cyclic uniaxial strain at a frequency of 0.5 Hz. Cell reorientation, quantified as a percentage of cells that reoriented 90° ± 30° relative to the strain direction, was decreased for nesprin-1 deficient cells (Nes-1) (*p* < 0.01). **b** The number of focal adhesions (FAs) increased in Nes-1 (*p* < 0.05). **c** Phase contrast images and traction stress maps. Scale bar = 200 μm. **d** Strain energy and **e** nuclear height were increased in Nes-1 (*p* < 0.05). A similar increase was observed with nonmuscle myosin II inhibitor (blebbistatin, Bleb) treatment. Adapted and reprinted with permission from [36]

An attempt to directly apply mechanical force to an isolated nucleus via nesprin-1 link demonstrated an induced reinforcement of the connection between the LINC complex and lamin A/C [34]. In this study with fibroblasts, the stretching of nesprin-1 triggered the Src-dependent phosphorylation of emerin, thus changing the nature of its association with lamin and leading to nucleus stiffening. In the cyclic mechanical stretching of myoblasts, disruption of the LINC complex impaired the mechanical stimulation of terminal myogenic differentiation [35]. Chancellor et al. [36] tested the role of the LINC complex in adhesion, migration, and orientation of human vascular endothelial cells (HUVECs) under uniaxial strain and showed that cells were unable to reorient in response to the strain in the presence of nesprin-1 silencing (Fig. 3). They also observed that nesprin-1 deficient cells displayed a larger number of focal adhesions and higher nuclear heights. Their proposed model suggested that the actomyosin tension on the nucleus is exerted and balanced by connections via nesprin linkage: when this nesprin connection is disrupted, the tension can be balanced with increased focal adhesions. In another study, endothelial cells with nesprin-1 knockdown displayed a decreased nuclear width and an increase in nucleus strain when exposed to uniaxial stretch loading [37]. This suggested that silencing nesprin-1 could release the nucleus from the tension of F-actin filament, thus allowing for deformation before stretching. Also, fibroblastic cells manipulated with nesprin-1 siRNA showed decreased cell elongation under cyclic stretch [38].

Some studies tested the role of the LINC complex without dynamic external mechanical loading (fluid shear or stretch) but on substrates with varying stiffness. It was demonstrated that the LINC complex could facilitate the regulation of genome-wide transcriptional changes in fibroblasts in response to substrate rigidity, but at the same time interfering with the LINC complex did not attenuate the sensitivity of the nuclear shape change caused by the substrate rigidity [39]. For human muscle precursor cells, intact lamin and nesprin-1 were required to enable cells to adapt their intracellular tension to the rigidity of the ECM [40].

## LINC complex regulation of MSC function and fate

MSCs have been of significant interest as they can serve as a promising cell source for tissue engineering and regenerative medicine due to their multi-lineage potential. Now, we will delve into the main focus of this review, a potential role of the LINC complex in MSC behavior, lineage commitment, and differentiation. The regulatory role of the LINC complex will be discussed for cases in the absence and presence of external mechanical loading.

### LINC complex control of MSC behavior without mechanical loading

**Fig. 4:**
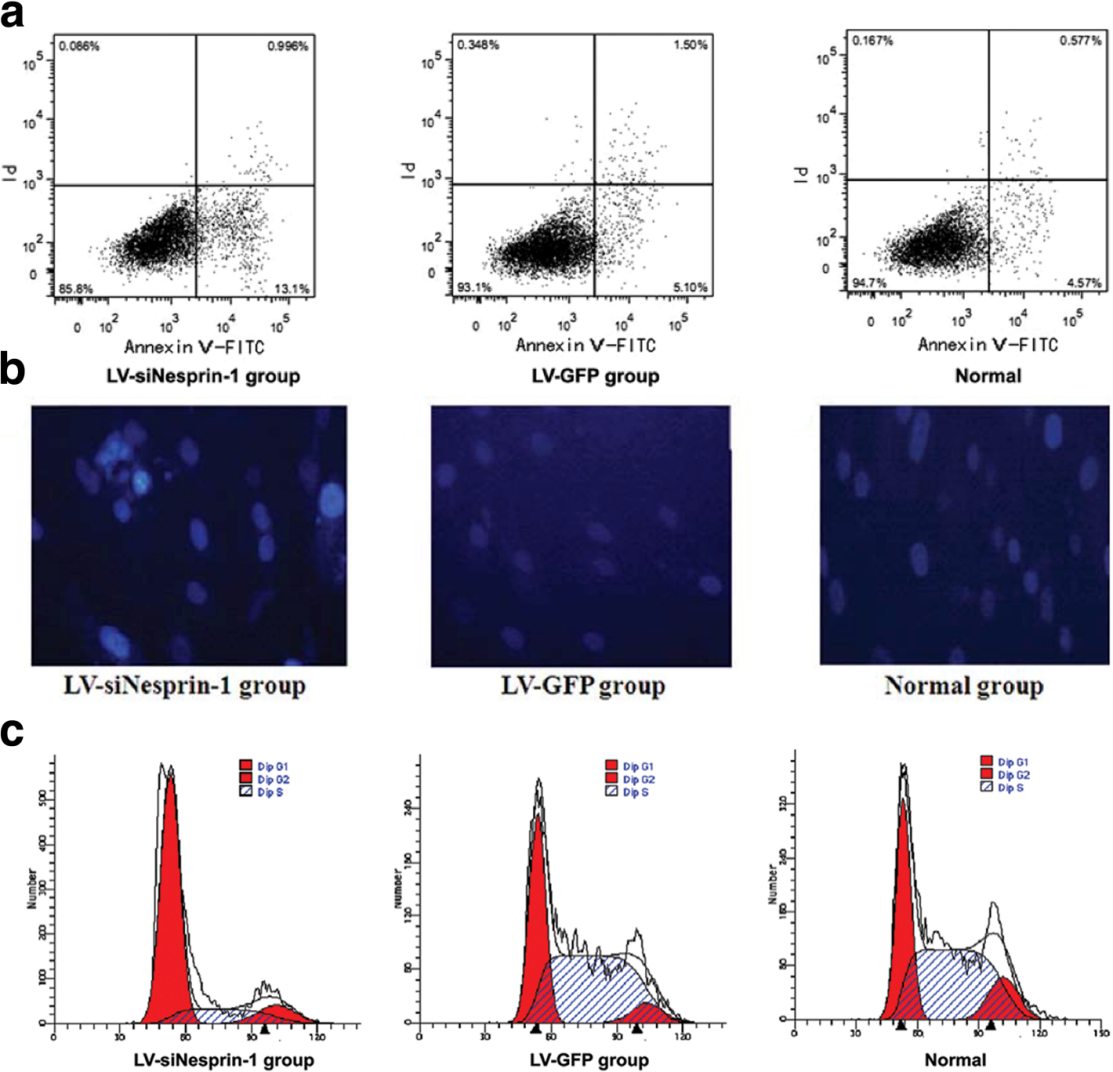
Nesprin-1 plays a regulatory role in the proliferation, apoptosis, and myogenesis of MSCs. **a** MSCs were transfected with either LV-siNesprin-1 or LV-GFP and cell apoptosis was analyzed using flow cytometry. Apoptosis was the greatest in the LV-siNesprin-1 MSCs. **b** Nuclear morphology was observed 72 h after transfection (DAPI). The LIV-siNesprin-1 group exhibited nuclear fusion and fragmentation. **c** Differentiation of the LIV-siNesprin-1 group was significantly slower than the LV-GFP and Normal groups; majority of the cells remained in the G0/G1 phase of the cell cycle. Adapted and reprinted with permission from [41]

Several studies have looked into the role of the LINC complex and its associated proteins in MSC function in the absence of external mechanical inputs. As shown for other cell types, the LINC complex can be involved in various aspects of MSC morphology and function. In a study by Yang et al. [41], rat bone marrow-derived MSCs (BMSCs) were transfected with a lentiviral vector expressing siRNA targeting nesprin-1. With transfection, MSCs exhibited a decrease in cell proliferation and an increase in apoptosis (Fig. 4a,b). Moreover, nesprin-1 siRNA significantly altered nuclear morphology, frequently resulting in cell fusion or fragmentation. In another study with rat BMSCs [42], cells with SUN1-depleted nuclei displayed reduced nucleus stiffness and substantial reduction in osteopontin-induced cell migration.

**Fig. 5:**
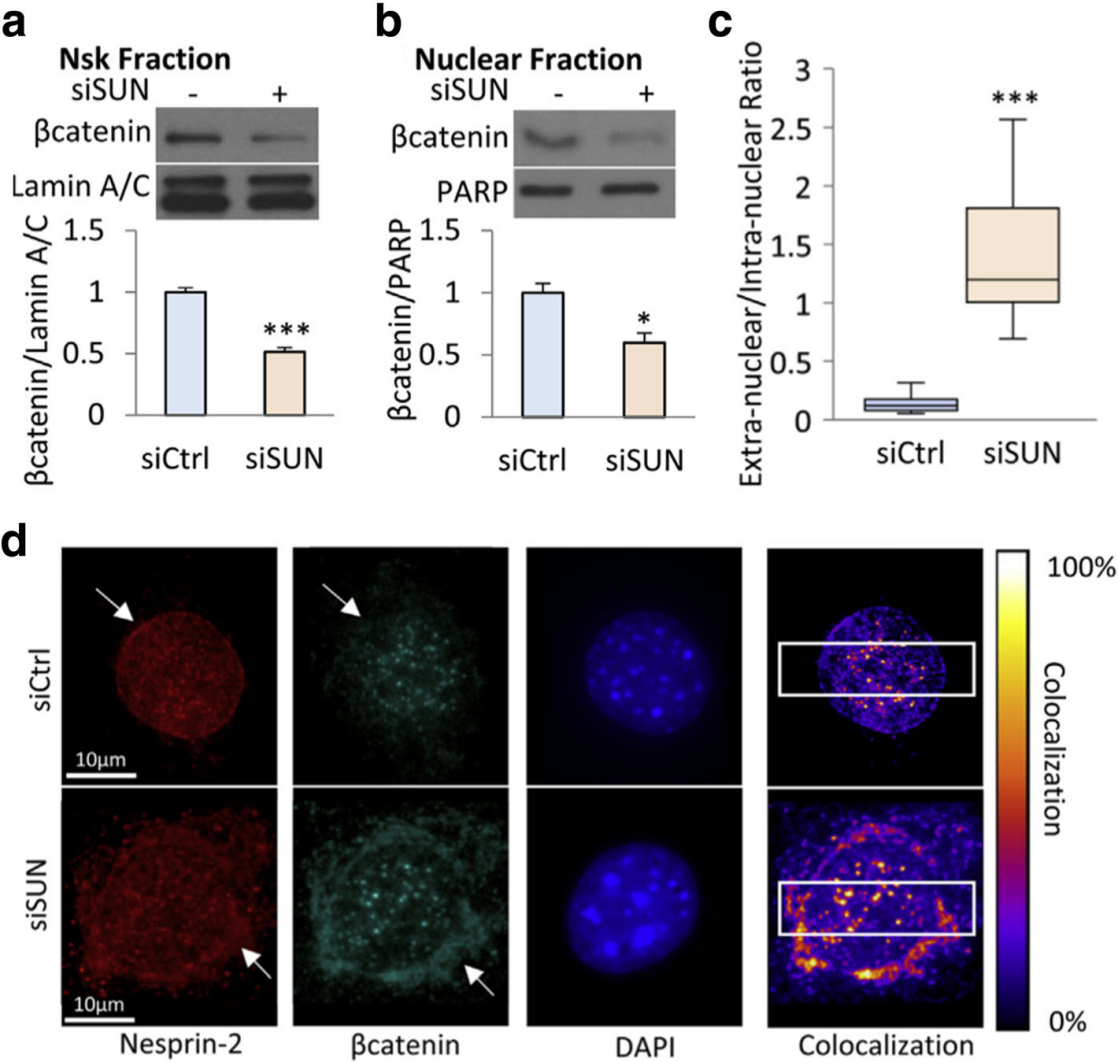
Co-deletion of SUN1 and SUN2 in MSCs disrupts β-catenin association with the nucleoskeleton. SUN1 and SUN2 were depleted via siRNA (siSUN) (**a**, **b**) siSUN decreased β-catenin-nucleoskeleton (Nsk) association and nuclear β-catenin level, as relative to the control (siCtrl). **c**, **d** In siSUN-treated cells, nesprin-2 and β-catenin were primarily located outside the nucleus; however, they were inside the nucleus in control siCtrl cells (white arrows). *: *p* < 0.05, ***: *p* < 0.001. Adapted and reprinted with permission from [46]

Increasing evidence proposes that the LINC complex may play an important role in MSC fate decision and phenotypic commitment. As it was suggested that a correct positioning of nuclei via nesprin-1 is required for myotube formation and resultant muscle function [43], it may be probable that nesprin plays a role in MSC myogenic differentiation. Indeed, myoblast differentiation was substantially deteriorated in nesprin-1 deficient BMSCs [41] (Fig. 4c). In addition to myogenesis, nuclear envelope proteins have been shown to regulate MSC adipocytic commitment. The Wnt/β-catenin signaling has been established to mediate MSC adipogenesis: nuclear translocation of β-catenin allows for the downregulation of adipogenic transcription factors leading to decreased adipocytic commitment [44]. The β-catenin entrance into the nucleus is achieved through interacting with nuclear pore complexes (NPCs), and direct coordination of β-catenin with the LINC complex can enhance the β-catenin membrane localization for nuclear import via NPCs [45]. Accordingly, the co-deletion of SUN1 and SUN2 and the subsequent untethering of nesprin-2 from the nuclear membrane in MSCs significantly diminished β-catenin nuclear surface localization and β-catenin levels obtained from the soluble nuclear fraction [46] (Fig. 5).

### LINC complex control of MSC behavior under mechanical loading

Understanding the mechanisms by which the mechanical microenvironments regulate MSC function and developmental fate is important for functional tissue engineering. Recent research has explored how MSCs perceive and respond to mechanical loading environments, particularly focusing on the nucleus and nuclear proteins. In a study utilizing Rho-associated protein kinase (ROCK) inhibition to induce a loss of cytoskeletal tension in MSCs [47], ROCK inhibition decreased nuclear deformation under static stretch and abrogated nesprin-1 upregulation under dynamic stretch, suggesting the coordination of cytoskeletal tension and LINC complex function in MSC behavior.

**Fig. 6:**
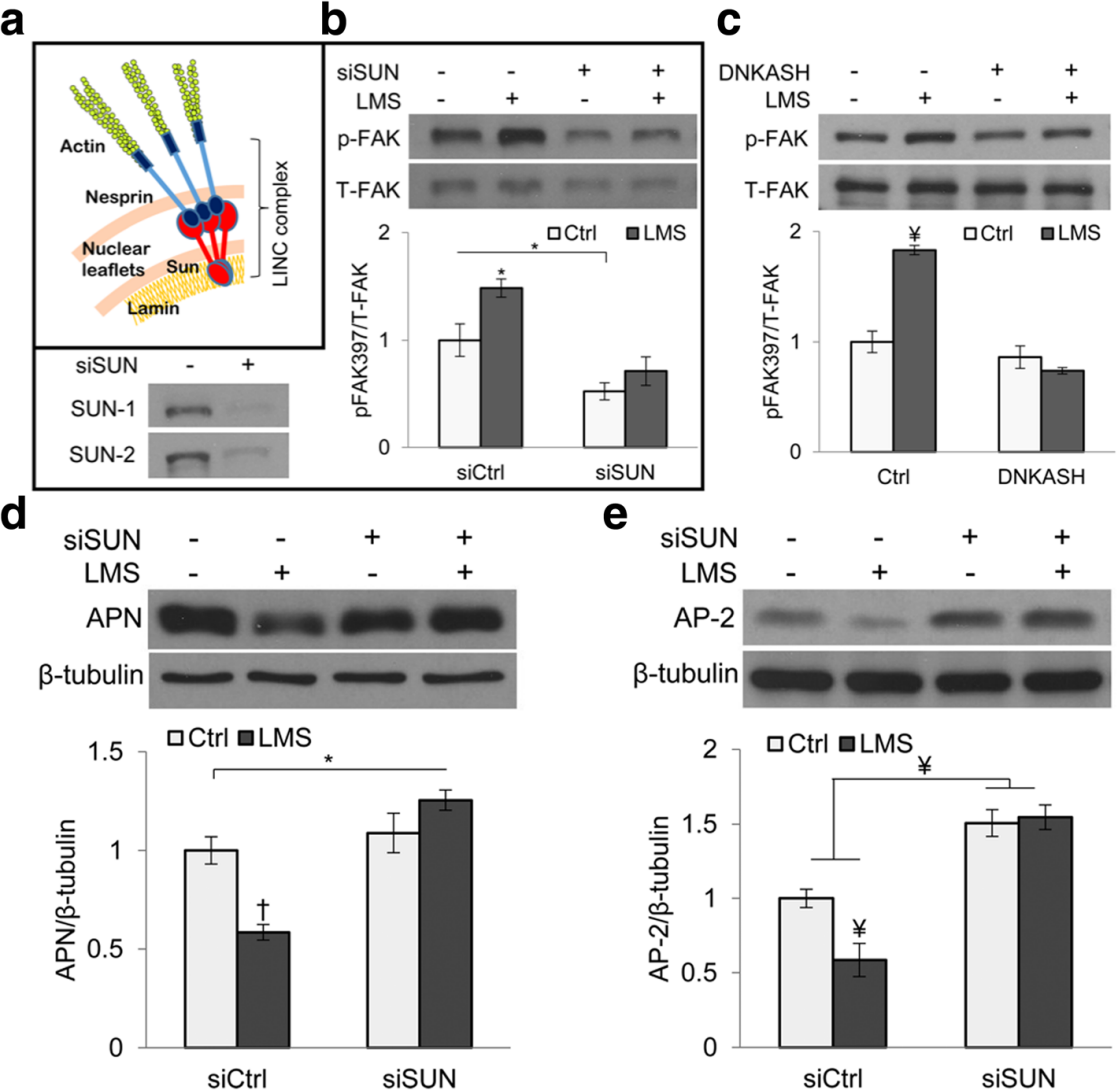
The LINC complex connection to actin cytoskeleton is necessary for low-magnitude signal-induced suppression of MSC adipogenesis. **a** Schematic of the LINC complex. **b**,**c** Decoupling the LINC complex via silencing SUN with siRNA (siSUN) or overexpressing dominant negative KASH (DNKASH) in MSCs disrupted low-magnitude signal (LMS)-induced focal adhesion kinase (FAK) activation. **d**, **e** In siSUN treated cells, the LMS inhibition of adipogenesis is impaired, as assessed by adipogenic markers, adiponectin (APN) and AP-2. *: *p* < 0.05; ¥: *p* < 0.01; †: *p* < 0.001. Adapted and reprinted with permission from [48]

Various studies have pointed to the role of mechanical loading in directing MSC fate, and recently the focus has been targeting the LINC complex as a key component of such phenomena. For example, in a study by Uzer et al. [48] on the inhibition of MSC adipogenesis by low- and high-magnitude mechanical signals (LMS and HMS, respectively), an intact LINC complex was necessary for the LMS function, but not for the HMS action (Fig. 6). The decreases in adipogenic markers (adiponectin and AP-2) in MSCs by LMS were abolished in the presence of siRNA of SUN. Despite the relatively well-established correlation between MSC adipogenesis vs. osteogenesis in response to mechanical environments [44, 49–51], the LINC complex regulation of MSC osteogenesis by mechanical loading has not been studied intensively. For myogenesis, while the LINC complex has been shown to mediate the mechanical myogenic direction for various myogenic cell lines, there are limited studies on the LINC regulation of the mechanical induction of MSC myogenesis. One study showed that treatment of MSCs with 5-azacytidine to induce in vitro cardiomyogenesis resulted in an increase in nesprin-1 expression [52]. Moreover, nesprin-1 expression was higher in the infarcted rat myocardium implanted with MSCs than in the non-implanted control group, suggesting the involvement of nesprin-1 in MSC differentiation into myocardial phenotype. While these studies suggest a functional association between LINC and MSC lineage commitment, the underlying mechanotransduction mechanisms remain to be fully explored.

**Fig. 7:**
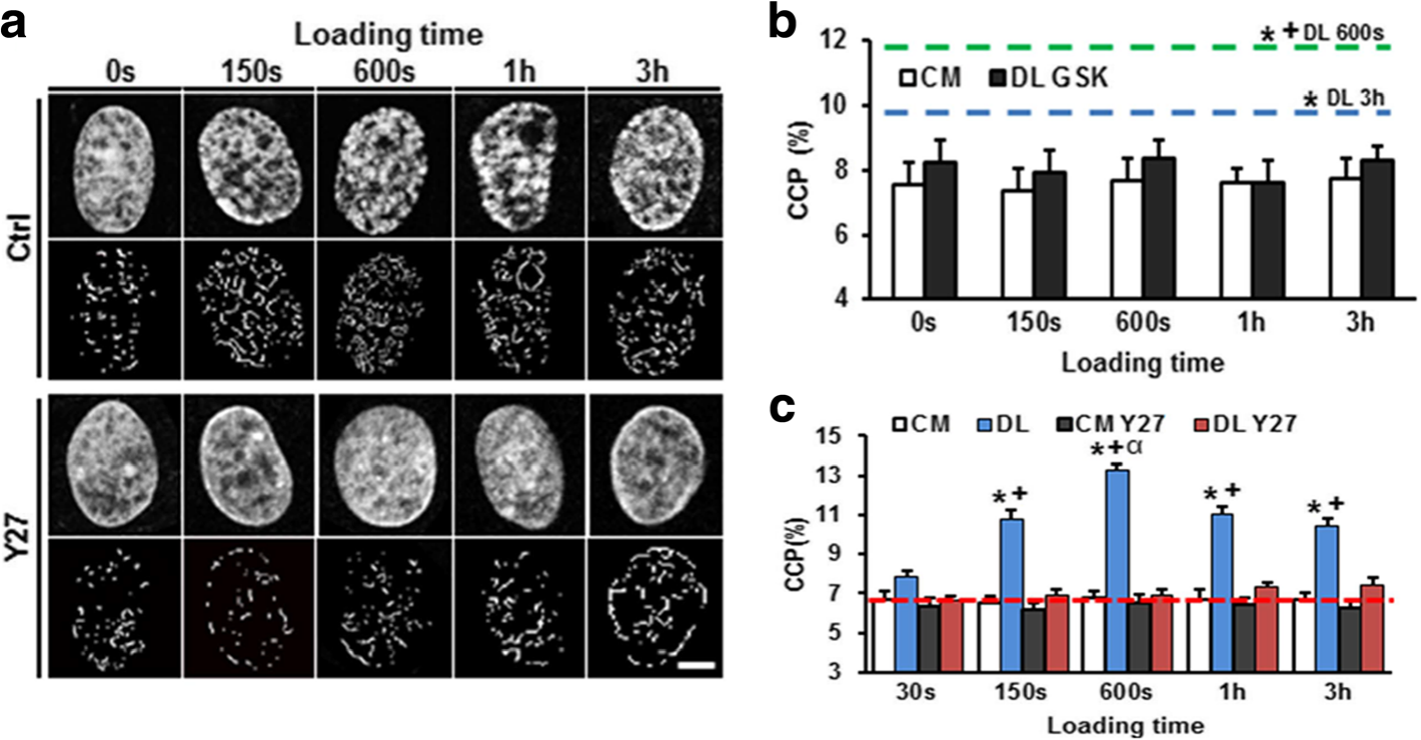
Dynamic tensile loading induces chromatin condensation in MSCs via the activation of histone modifying enzymes. **a** MSCs were seeded onto aligned nanofibrous scaffolds and exposed to dynamic tensile loading (3% strain at 1 Hz). DAPI-stained nuclei (top) and chromatin condensation parameter (CCP) image processing (bottom) for control and under ROCK inhibitor Y27. **b** Chromatin condensation (assessed by CCP) was increased by dynamic tensile loading (DL): green and blue lines. This was abrogated under GSK343, an inhibitor of histone methyltransferase EZH2. The control is treated with control media (CM). *: *p* < 0.05 vs. CM at 0 s, +: *p* < 0.05 vs. DL 3 h in CM. **c** DL induction of increase in CCP was impaired by ROCK inhibitor Y27. *: *p* < 0.05 vs. CM control, +: *p* < 0.05 vs. Y27, α: *p* < 0.05 vs. 150 s. Adapted and reprinted with permission from [55]

How MSCs translate mechanical signals into modifications at the genomic level is not fully understood, either. For embryonic stem cells (ESCs), it is known that the chromatin structure becomes gradually more condensed following differentiation [53]. Clusters of condensation can be associated with regions of transcriptionally inactive genes, while decondensed euchromatin regions allow for the access of their binding sites and activate lineage-specific gene expression [54]. Interestingly, for MSCs, dynamic mechanical loading could induce rapid calcium-dependent chromatin condensation in the absence of exogenous differentiation factors [55] (Fig. 7). In this study, a load-dependent persistence in chromatin condensation in MSCs required the continued activity of histone methyltransferases and acetylases after the cessation of loading, implying the mechanical memory effect of previous loading events. Considering the potential physical interconnection between the LINC complex and nuclear chromatin as described above [22, 23], further investigation into whether the LINC complex behaves as an intermediate in the observed load-induced chromatin condensation and lineage specification is required.

**Fig. 8:**
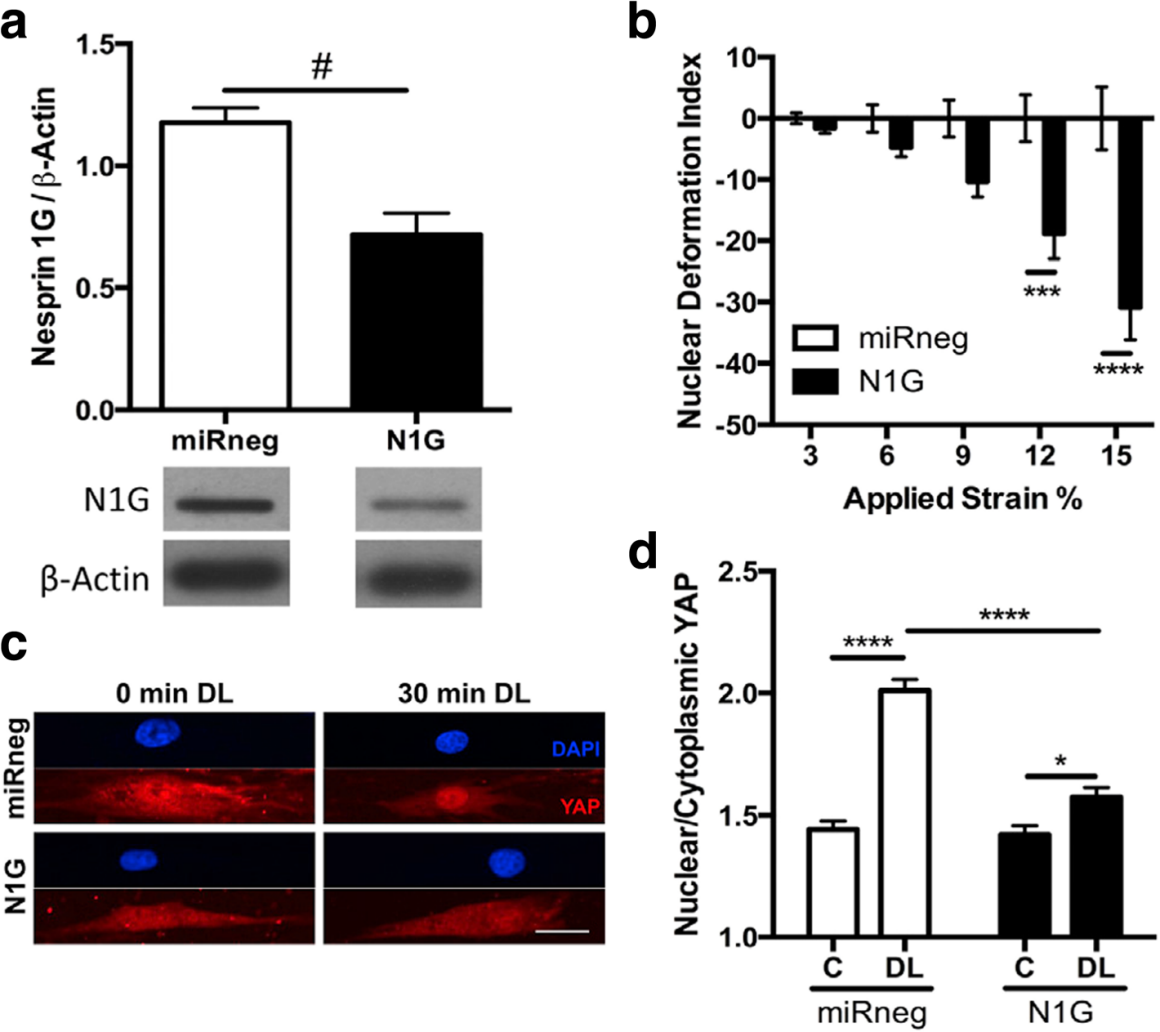
YAP nuclear translocation in response to dynamic tensile loading in MSCs requires strain transfer to the nucleus via nesprin-1G. MSCs were seeded on aligned nanofibers and exposed to dynamic tensile loading (3% strain at 1 Hz). **a** Nesprin-1 giant knockdown (N1G) compared to cells treated with non-targeting miRNA (miRneg). **b** The nuclear deformation index quantified for the N1G group compared to the control at varying strains applied. **c** YAP- and DAPI-stained nuclei with or without 30 min of dynamic tensile loading (DL). Scale bar = 25 μm. **d** The ratio of nuclear to cytoplasmic YAP content was increased by DL (control miRneg), which effect was suppressed by nesprin-1 knockdown (N1G). #: *p* < 0.05, *: *p* < 0.05, ***: *p* < 0.001, ****: *p* < 0.0001. Reprinted with permission from [57]

MSCs perceive the stiffness of ECM, cell micropatterning size, or resultant changes in cell shape via the activation and regulation of key nuclear transcription factors, one of which is Yes-associated protein (YAP)/transcriptional coactivator with PDZ-binding motif (TAZ) [56]. YAP/TAZ nuclear localization in MSCs is known to be Rho GTPase-dependent, which regulates the formation of actin stress fibers in response to ECM stiffness and cell spreading. Interestingly, similar to the static substrate control of YAP/TAZ, dynamic tensile stretching of MSCs could induce the nuclear translocalization of YAP [57] (Fig. 8). Further, this activity in YAP under stretch loading may require strain transfer to the nucleus via nesprin-1G, as evidenced by the decrease in nuclear/cytoplasmic YAP content under stretch with nesprin-1G knockdown. On the other hand, the mechanism by which YAP participates in the crosstalk with nesprin in the cytoplasmic area under static condition (before loading) and how YAP and nesprin coordination will occur under dynamic loading can be investigated further.

## Future perspective

Mechanosensitive proteins found at the NE have increasingly been shown to play a key part in modulating the biomechanical information transfer between the cell surface, cytoskeletal structures, and the nucleus. For MSCs, although it is well established that cells rely on mechanical cues to reorganize their internal physical structures and regulate various signaling pathways, there remains much to be explored and understood about the relationship between nuclear mechanics and stem cell behavior including fate decision. For example, what is the molecular mechanism enabling MSCs to perceive the external mechanical environment and propagate the force signal to the nucleus, and how do NE proteins such as the LINC complex and the components, nesprin and SUN, further transmit the information to internal nuclear machinery? Moreover, in what way do the LINC proteins physically interact with chromatin and transcriptional regulatory factors to mediate changes in gene expression and direct stem cell differentiation? What role does the LINC complex play in modulating MSC mechanical adaptivity and memory effect, and how do these influence load-controlled MSC fate and long-term phenotype commitment? Additionally, relative to external mechanical loading studies, there lacks research testing the involvement of the LINC complex in MSC function and lineage commitment on substrates of varying stiffness or geometries. Such information may allow for the development and optimization of material-based devices for diagnostics, tissue engineering, and regenerative medicine. While considerable progress has been made in the past decades in identifying the mechanisms by which MSCs respond and adapt to external mechanical loading environments, studies should take into more consideration on the nucleus, particularly the LINC complex and associated nesprin and SUN, as an integral component of the cellular mechanosensory circuit that regulates MSC function, lineage commitment, and terminal differentiation.

## Data Availability

Not applicable.

## Abbreviations

BMSC: Bone-marrow derived mesenchymal stem cell
CH: Calponin homology
ECM: Extracellular matrix
EDMD: Emery-Dreifuss muscular dystrophy
ESC: Embryonic stem cell
FRET: Fluorescence resonance energy transfer
HMS: High-magnitude mechanical signal
HUVEC: Human vascular endothelial cell
IF: Intermediate filament
INM: Inner nuclear membrane
KASH: Klarsicht/ANC-1/Syne homology
LINC: Linker of nucleoskeleton and cytoskeleton
LMS: Low-magnitude mechanical signal
LPA: Lysophosphatidic acid
MSC: Mesenchymal stem cell
NE: Nuclear envelope
nesprin: Nuclear envelope spectrin-repeat protein
NPC: Nuclear pore complex
ONM: Outer nuclear membrane
PNS: Perinuclear space
ROCK: Rho-associated protein kinase
TAN lines: Transmembrane actin-associated nuclear lines
TAZ: Transcriptional coactivator with PDZ-binding motif
YAP: Yes-associated protein

## References

[1] Wang N, Butler JP, Ingber DE (1993). Mechanotransduction across the cell surface and through the cytoskeleton. Science..

[2] Geiger B, Yamada KM (2011). Molecular architecture and function of matrix adhesions. Cold Spring Harb Perspect Biol.

[3] Maniotis AJ, Chen CS, Ingber DE (1997). Demonstration of mechanical connections between integrins, cytoskeletal filaments, and nucleoplasm that stabilize nuclear structure. Proc Natl Acad Sci U S A.

[4] Stewart CL, Roux KJ, Burke B (2007). Blurring the boundary: the nuclear envelope extends its reach. Science..

[5] Roux KJ, Crisp ML, Liu Q, Kim D, Kozlov S, Stewart CL, Burke B (2009). Nesprin 4 is an outer nuclear membrane protein that can induce kinesin-mediated cell polarization. Proc Natl Acad Sci U S A.

[6] Wang N, Tytell JD, Ingber DE (2009). Mechanotransduction at a distance: mechanically coupling the extracellular matrix with the nucleus. Nat Rev Mol Cell Biol..

[7] Mellad JA, Warren DT, Shanahan CM (2011). Nesprins LINC the nucleus and cytoskeleton. Curr Opin Cell Biol.

[8] Uzer G, Fuchs RK, Rubin J, Thompson WR (2016). Concise review: plasma and nuclear membranes convey mechanical information to regulate mesenchymal stem cell lineage. Stem Cells.

[9] Lombardi ML, Jaalouk DE, Shanahan CM, Burke B, Roux KJ, Lammerding J (2011). The interaction between nesprins and sun proteins at the nuclear envelope is critical for force transmission between the nucleus and cytoskeleton. J Biol Chem.

[10] Zhang Q, Skepper JN, Yang F, Davies JD, Hegyi L, Roberts RG, Weissberg PL, Ellis JA, Shanahan CM (2001). Nesprins: a novel family of spectrin-repeat-containing proteins that localize to the nuclear membrane in multiple tissues. J Cell Sci.

[11] Starr DA, Han M (2003). ANChors away: an actin based mechanism of nuclear positioning. J Cell Sci.

[12] Crisp M, Liu Q, Roux K, Rattner JB, Shanahan C, Burke B, Stahl PD, Hodzic D (2006). Coupling of the nucleus and cytoplasm: role of the LINC complex. J Cell Biol.

[13] Zhang Q, Ragnauth CD, Skepper JN, Worth NF, Warren DT, Roberts RG, Weissberg PL, Ellis JA, Shanahan CM (2005). Nesprin-2 is a multi-isomeric protein that binds Lamin and emerin at the nuclear envelope and forms a subcellular network in skeletal muscle. J Cell Sci.

[14] Wilhelmsen K, Litjens SH, Kuikman I, Tshimbalanga N, Janssen H, van den Bout I, Raymond K, Sonnenberg A (2005). Nesprin-3, a novel outer nuclear membrane protein, associates with the cytoskeletal linker protein plectin. J Cell Biol.

[15] Morimoto A, Shibuya H, Zhu X, Kim J, Ishiguro KI, Han M, Watanabe Y (2012). A conserved KASH domain protein associates with telomeres, SUN1, and dynactin during mammalian meiosis. J Cell Biol.

[16] Arsenovic PT, Ramachandran I, Bathula K, Zhu R, Narang JD, Noll NA, Lemmon CA, Gundersen GG, Conway DE (2016). Nesprin-2G, a component of the nuclear LINC complex, is subject to myosin-dependent tension. Biophys J.

[17] Chang W, Antoku S, Östlund C, Worman HJ, Gundersen GG (2015). Linker of nucleoskeleton and cytoskeleton (LINC) complex-mediated actin-dependent nuclear positioning orients centrosomes in migrating myoblasts. Nucleus..

[18] Koszul R, Kim KP, Prentiss M, Kleckner N, Kameoka S (2008). Meiotic chromosomes move by linkage to dynamic actin cables with transduction of force through the nuclear envelope. Cell..

[19] Warren DT, Tajsic T, Porter LJ, Minaisah RM, Cobb A, Jacob A, Rajgor D, Zhang QP, Shanahan CM (2015). Nesprin-2-dependent ERK1/2 compartmentalisation regulates the DNA damage response in vascular smooth muscle cell ageing. Cell Death Diff.

[20] Lei K, Zhu X, Xu R, Shao C, Xu T, Zhuang Y, Han M (2012). Inner nuclear envelope proteins SUN1 and SUN2 play a prominent role in the DNA damage response. Curr Biol.

[21] Lawrence KS, Tapley EC, Cruz VE, Li Q, Aung K, Hart KC, Schwartz TU, Starr DA, Engebrecht J (2016). LINC complexes promote homologous recombination in part through inhibition of nonhomologous end joining. J Cell Biol.

[22] Rubin J, Sen B (2017). Actin up in the nucleus: regulation of actin structures modulates mesenchymal stem cell differentiation. Trans Am Clin Climatol Assoc.

[23] Rashmi RN, Eckes B, Glöckner G, Groth M, Neumann S, Gloy J, Sellin L, Walz G, Schneider M, Karakesisoglou I, Eichinger L (2012). The nuclear envelope protein Nesprin-2 has roles in cell proliferation and differentiation during wound healing. Nucleus..

[24] Zhang X, Lei K, Yuan X, Wu X, Zhuang Y, Xu T, Xu R, Han M (2009). SUN1/2 and Syne/Nesprin-1/2 complexes connect centrosome to the nucleus during neurogenesis and neuronal migration in mice. Neuron..

[25] Luxton GG, Gomes ER, Folker ES, Vintinner E, Gundersen GG (2010). Linear arrays of nuclear envelope proteins harness retrograde actin flow for nuclear movement. Science..

[26] Wu J, Kent IA, Shekhar N, Chancellor TJ, Mendonca A, Dickinson RB, Lele TP (2014). Actomyosin pulls to advance the nucleus in a migrating tissue cell. Biophys J.

[27] Méjat A, Misteli T (2010). LINC complexes in health and disease. Nucleus..

[28] Stephens PJ, Tarpey PS, Davies H, Van Loo P, Greenman C, Wedge DC, Nik-Zainal S, Martin S, Varela I, Bignell GR, Yates LR, Papaemmanuil E, Beare D, Butler A, Cheverton A, Gamble J, Hinton J, Jia M, Jayakumar A, Jones D, Latimer C, Lau KW, McLaren S, McBride DJ, Menzies A, Mudie L, Raine K, Rad R, Chapman MS, Teague J, Easton D, Langerød A, Lee MT, Shen CY, Tee BT, Huimin BW, Broeks A, Vargas AC, Turashvili G, Martens J, Fatima A, Miron P, Chin SF, Thomas G, Boyault S, Mariani O, Lakhani SR, van de Vijver M, van 't Veer L, Foekens J, Desmedt C, Sotiriou C, Tutt A, Caldas C, Reis-Filho JS, Aparicio SA, Salomon AV, Børresen-Dale AL, Richardson AL, Campbell PJ, Futreal PA, Stratton MR, Oslo Breast Cancer Consortium (OSBREAC) (2012). The landscape of cancer genes and mutational processes in breast cancer. Nature.

[29] Sjöblom T, Jones S, Wood LD, Parsons DW, Lin J, Barber TD, Mandelker D, Leary RJ, Ptak J, Silliman N, Szabo S (2006). The consensus coding sequences of human breast and colorectal cancers. Science..

[30] Doherty JA, Rossing MA, Cushing-Haugen KL, Chen C, Van Den Berg DJ, Wu AH, Pike MC, Ness RB, Moysich K, Chenevix-Trench G, Beesley J (2010). ESR1/SYNE1 polymorphism and invasive epithelial ovarian cancer risk: an ovarian Cancer association consortium study. Cancer Epidemiol Biomark Prev.

[31] Han Y, Wang L, Yao QP, Zhang P, Liu B, Wang GL, Shen BR, Cheng B, Wang Y, Jiang ZL, Qi YX (1853). Nuclear envelope proteins Nesprin2 and LaminA regulate proliferation and apoptosis of vascular endothelial cells in response to shear stress. Biochim Biophys Acta.

[32] Morgan JT, Pfeiffer ER, Thirkill TL, Kumar P, Peng G, Fridolfsson HN, Douglas GC, Starr DA, Barakat AI (2011). Nesprin-3 regulates endothelial cell morphology, perinuclear cytoskeletal architecture, and flow-induced polarization. Mol Biol Cell.

[33] Chambliss AB, Khatau SB, Erdenberger N, Robinson DK, Hodzic D, Longmore GD, Wirtz D (2013). The LINC-anchored actin cap connects the extracellular milieu to the nucleus for ultrafast mechanotransduction. Sci Rep.

[34] Guilluy C, Osborne LD, Van Landeghem L, Sharek L, Superfine R, Garcia-Mata R, Burridge K (2014). Isolated nuclei adapt to force and reveal a mechanotransduction pathway in the nucleus. Nat Cell Biol.

[35] Brosig M, Ferralli J, Gelman L, Chiquet M, Chiquet-Ehrismann R (2010). Interfering with the connection between the nucleus and the cytoskeleton affects nuclear rotation, mechanotransduction and myogenesis. Int J Biochem Cell Biol.

[36] Chancellor TJ, Lee J, Thodeti CK, Lele T (2010). Actomyosin tension exerted on the nucleus through nesprin-1 connections influences endothelial cell adhesion, migration, and cyclic strain-induced reorientation. Biophys J.

[37] Anno T, Sakamoto N, Sato M (2012). Role of nesprin-1 in nuclear deformation in endothelial cells under static and uniaxial stretching conditions. Biochem Biophys Res Commun.

[38] Sakamoto N, Ogawa M, Sadamoto K, Takeuchi M, Kataoka N (2017). Mechanical role of nesprin-1-mediated nucleus-actin filament binding in cyclic stretch-induced fibroblast elongation. Cell Mol Bioeng.

[39] Alam SG, Zhang Q, Prasad N, Li Y, Chamala S, Kuchibhotla R, Birendra KC, Aggarwal V, Shrestha S, Jones AL, Levy SE (2016). The mammalian LINC complex regulates genome transcriptional responses to substrate rigidity. Sci Rep.

[40] Schwartz C, Fischer M, Mamchaoui K, Bigot A, Lok T, Verdier C, Duperray A, Michel R, Holt I, Voit T, Quijano-Roy S (2017). Lamins and nesprin-1 mediate inside-out mechanical coupling in muscle cell precursors through FHOD1. Sci Rep.

[41] Yang W, Zheng H, Wang Y, Lian F, Hu Z, Xue S (2013). Nesprin-1 plays an important role in the proliferation and apoptosis of mesenchymal stem cells. Int J Mol Med.

[42] Liu Lingling, Luo Qing, Sun Jinghui, Song Guanbin (2019). Cytoskeletal control of nuclear morphology and stiffness are required for OPN-induced bone-marrow-derived mesenchymal stem cell migration. Biochemistry and Cell Biology.

[43] Espigat-Georger A, Dyachuk V, Chemin C, Emorine L, Merdes A (2016). Nuclear alignment in myotubes requires centrosome proteins recruited by nesprin-1. J Cell Sci.

[44] Sen B, Xie Z, Case N, Ma M, Rubin C, Rubin J (2008). Mechanical strain inhibits adipogenesis in mesenchymal stem cells by stimulating a durable β-catenin signal. Endocrinology..

[45] Liu Q, Pante N, Misteli T, Elsagga M, Crisp M, Hodzic D, Burke B, Roux KJ (2007). Functional association of Sun1 with nuclear pore complexes. J Cell Biol.

[46] Uzer G, Bas G, Sen B, Xie Z, Birks S, Olcum M, McGrath C, Styner M, Rubin J (2018). Sun-mediated mechanical LINC between nucleus and cytoskeleton regulates βcatenin nuclear access. J Biomech.

[47] Driscoll TP, Shurden ZE, Heo S, Mauck, RL. Cytoskeletal tension is required for dynamic tensile loading induced alterations in mesenchymal stem cell shape and nuclear connectivity. 2013 Orthopaedic Research Society Annual Meeting. Paper No: 0088.

[48] Uzer G, Thompson WR, Sen B, Xie Z, Yen SS, Miller S, Bas G, Styner M, Rubin CT, Judex S, Burridge K (2015). Cell mechanosensitivity to extremely low-magnitude signals is enabled by a LINCed nucleus. Stem Cells.

[49] David V, Martin A, Lafage-Proust MH, Malaval L, Peyroche S, Jones DB, Vico L, Guignandon A (2007). Mechanical loading down-regulates peroxisome proliferator-activated receptor γ in bone marrow stromal cells and favors osteoblastogenesis at the expense of adipogenesis. Endocrinology.

[50] Lee JS, Ha L, Park JH, Lim JY (2012). Mechanical stretch suppresses BMP4 induction of stem cell adipogenesis via upregulating ERK but not through downregulating Smad or p38. Biochem Biophys Res Commun.

[51] Poudel I, Menter DE, Lim JY (2012). Directing cell function and fate via micropatterning: role of cell patterning size, shape, and interconnectivity. Biomed Eng Lett.

[52] Yang W, Zheng H, Wang Y, Lian F, Hu Z, Xue S (2015). Nesprin 1 has key roles in the process of mesenchymal stem cell differentiation into cardiomyocyte like cells in vivo and in vitro. Mol Med Rep.

[53] Gaspar-Maia A, Alajem A, Meshorer E, Ramalho-Santos M (2011). Open chromatin in pluripotency and reprogramming. Nat Rev Mol Cell Biol.

[54] Schneider R, Grosschedl R (2007). Dynamics and interplay of nuclear architecture, genome organization, and gene expression. Genes Dev.

[55] Heo SJ, Thorpe SD, Driscoll TP, Duncan RL, Lee DA, Mauck RL (2015). Biophysical regulation of chromatin architecture instills a mechanical memory in mesenchymal stem cells. Sci Rep.

[56] Dupont S, Morsut L, Aragona M, Enzo E, Giulitti S, Cordenonsi M, Zanconato F, Le Digabel J, Forcato M, Bicciato S, Elvassore N, Piccolo S (2011). Role of YAP/TAZ in mechanotransduction. Nature..

[57] Driscoll TP, Cosgrove BD, Heo SJ, Shurden ZE, Mauck RL (2015). Cytoskeletal to nuclear strain transfer regulates YAP signaling in mesenchymal stem cells. Biophys J.

